# Effect of SWCNT-Tuball Paper on the Lightning Strike Protection of CFRPs and Their Selected Mechanical Properties

**DOI:** 10.3390/ma14113140

**Published:** 2021-06-07

**Authors:** Kamil Dydek, Anna Boczkowska, Rafał Kozera, Paweł Durałek, Łukasz Sarniak, Małgorzata Wilk, Waldemar Łogin

**Affiliations:** 1 Faculty of Materials Science and Engineering, Warsaw University of Technology, ul. Wołoska 141, 02-507 Warsaw, Poland; anna.boczkowska@pw.edu.pl (A.B.); rafal.kozera@pw.edu.pl (R.K.); lukasz.sarniak@pw.edu.pl (Ł.S.); 2 TMBK Partners Sp. z o.o., ul. Pawińskiego 5A, 02-106 Warsaw, Poland; pawel.duralek@tmbk.pl; 3 Polskie Zakłady Lotnicze Sp. z o.o.—PZL Mielec A Lockheed Martin Company, ul. Wojska Polskiego 3, 39-300 Mielec, Poland; malgorzata.wilk@lmco.com (M.W.); waldemar.login@lmco.com (W.Ł.)

**Keywords:** carbon nanotubes, Tuball paper, carbon fibre reinforced polymers, electrical conductivity, impact resistance, lightning strike protection, phased array

## Abstract

The main aim of this work was the investigation of the possibility of replacing the heavy metallic meshes applied onto the composite structure in airplanes for lightning strike protection with a thin film of Tuball single-wall carbon nanotubes in the form of ultra-light, conductive paper. The Tuball paper studied contained 75 wt.% or 90 wt.% of carbon nanotubes and was applied on the top of carbon fibre reinforced polymer before fabrication of flat panels. First, the electrical conductivity, impact resistance and thermo-mechanical properties of modified laminates were measured and compared with the reference values. Then, flat panels with selected Tuball paper, expanded copper foil and reference panels were fabricated for lightning strike tests. The effectiveness of lightning strike protection was evaluated by using the ultrasonic phased-array technique. It was found that the introduction of Tuball paper on the laminates surface improved both the surface and the volume electrical conductivity by 8800% and 300%, respectively. The impact resistance was tested in two directions, perpendicular and parallel to the carbon fibres, and the values increased by 9.8% and 44%, respectively. The dynamic thermo-mechanical analysis showed higher stiffness and a slight increase in glass transition temperature of the modified laminates. Ultrasonic investigation after lightning strike tests showed that the effectiveness of Tuball paper is comparable to expanded copper foil.

## 1. Introduction

Carbon Fibre Reinforced Polymers (CFRPs) are currently widely used as structural materials in many industries such as aerospace, automotive, marine, civil engineering, or sporting goods [1]. This is due to their very high strength and stiffness in relation to mass, which makes them extremely competitive with their metal counterparts [2]. Very good mechanical properties result from excellent properties of carbon fibres, which are also characterized by stable thermo-physical properties even at an elevated temperature. However, CFRPs have one main disadvantage, i.e., low electrical conductivity, too low for e.g., lightning strike protection (LSP) of airplanes or wind turbines [3,4]. The LSP principle is to discharge an electrical charge as quickly as possible through a conductive path located in the top layer of the composite structure. However, the amount of energy supplied is so large that a part of it is not discharged and gets deep into CFRPs, causing material damage [5]. A lightning strike can locally heat the typical commercial airplane structure to a temperature of 25,000 °C and generate extreme surge currents [6]. A high-intensity strike can damage or destroy components such as electrically controlled fuel valves, generators, power feeders, electrical distribution systems and finally result in a crash of the machine [7,8]. It is estimated that an aircraft can be struck by a lightning every 1000–10,000 h of flight, which translates into 1–2 lightning strikes (LS) per year. Therefore, providing adequate lightning protection for the airplane is crucial [9].

Improvement of CFRPs surface electrical conductivity and LSP can be achieved by introducing meshes or foils made of aluminum or copper on the surface of the CFRPs structures [10,11], which can lead to increasing the mass of the composite components [12]. Hence, a more promising solution seems to be the use of carbon particles [13,14], especially carbon nanotubes (CNTs) due to their properties, i.e., very high electrical conductivity, low density, and high aspect ratio [15], which are much higher than in the case of other carbon fillers, e.g., carbon black. The method of introducing carbon nanotubes as well as the manner of manufacturing composites, determine the level of conductivity of CFRPs. One solution is to add carbon nanotubes to the epoxy resin, then supersaturate the carbon reinforcement with the resulting mixture [16,17,18]. The production of composites by this method has one significant disadvantage. After introduction of carbon nanotubes to neat epoxy, the viscosity of the mixture increases drastically, hindering the flow of the resin between the carbon reinforcements. Other methods of introducing carbon nanotubes into the composite structure are growing them on carbon fibres [19] or spraying them onto carbon fibre reinforcement [20]. Carbon nanotubes can also be incorporated into the CFRPs structure in the form of intermediate products such as CNT-doped nonwovens which can be produced by electrospinning [21], melt-blown [22,23] as well as extrusion and pressing [24]. In these techniques, the nonwovens are made of concentrates that contain carbon nanotubes dispersed in a polymer matrix. Another approach is the production of nonwovens from neat polymers, followed by soaking them in a solution containing CNT, or spraying such a solution onto the surface of the nonwovens [25,26,27].

The next well-known method consists of introducing carbon nanotubes into the composite structure in the form of a buckypaper (BP), which is a thin film produced by filtering a highly concentrated solution of carbon nanotubes and appropriate solvent [28]. BP is most often based on multi-wall carbon nanotubes (MWCNTs) and can be applied on the surface [29] or as an interlayer of the composite [30]. It is known that both electrical and mechanical properties can be improved by introducing BP into the structure of CFRPs [31]. Moreover, BPs are also used to improve the mechanical properties of thermoplastics. Much higher modulus and failure strength values were obtained by making thermoplastic polyurethane/BP composites [32]. Besides, due to their very good electrical conductivity of 3000–5000 S/m [33], BPs are used in surface finishing of composite materials for electromagnetic interference (EMI) shielding or lightning strike protection [34]. In another work, numerical modeling confirmed the effectiveness of CFRPs modification by the introduction of BP as the top layer for lightning strike protection [35] The effects of modification of BP with silver particles have also been recorded [36]. The essential fact is that literature provides much less information about the application of single-walled carbon nanotubes (SWCNTs) in the BP form, mainly due to the high price of SWCNTs. However, there is a need for further research of this issue. Furthermore, little is known about applying Tuball paper (TP) for modification of CFRPs properties in order to improve LSP [37].

In the present study, layers of Tuball papers produced by OCSiAl Company from Tuball SWCNTs were placed on top of the CFRPs flat panels during their manufacturing. The main aim was to replace heavy copper mesh applied on the surface of composite structure in the airplane industry by a thin film of ultra-light, conductive Tuball paper. The Tuball papers used in this study are not yet commercial products. The use of TP based on SWCTNs produced by OCSiAl is not yet known in the literature but its application in the industry seems to be possible.

In the study, two types of Tuball paper differing in SWCNTs content, thickness and areal weight were studied in the context of the modification of CFRPs surfaces, volume electrical conductivity, impact, and dynamic thermomechanical properties. Moreover, the effect of expanded copper foil on lightning strike protection of composite flat panels were compared with the effects of the Tuball paper. The effectiveness of LSP was assessed visually and measured with the use of ultrasonic phased-array technique.

## 2. Materials and Methods

### 2.1. CFRP for Electrical and Mechanical Tests

Three types of CFRP panels (a reference one and two panels modified with Tuball papers) were produced for electrical and mechanical tests using commercial unidirectional carbon–epoxy prepreg with an aerial weight of 145 g/m^2^ and 35 wt.% of epoxy resin. The reference CFRP panel consisted of 14 layers of prepreg placed in the same direction with the layup [0]_14_. The CFRP panels were modified by the introduction of Tuball paper as their top layer before the panels manufacturing process. The characteristics of the Tuball papers used are shown in Table 1. Other information about the composition of the Tuball papers used in this study are confidential.

All laminates were made by the out-of-autoclave method, under a vacuum bag on a glass plate with a release film attached. In modified laminates, Tuball paper was placed as the first layer on the glass plate. After sticking all the layers together, the laminates were degassed (debulked) under vacuum and were then consolidated with the cure cycle recommended in the prepreg technical data sheet (TDS). In the first step, the laminates were heated up to 80 °C at the rate of 3 °C/min, then kept isothermally at this temperature for 60 min. In the second step, the temperature was raised to 130 °C and maintained for 120 min. In this case, the heating rate was also 3 °C/min. Then, the temperature was lowered to room temperature at the rate of 2 °C/min. At the end of the manufacturing process, the laminates were removed from the vacuum bag and they were cut to appropriate dimensions using a saw equipped with a diamond blade. The designation and thicknesses of the produced CFRPs are shown in Table 2.

In order to produce three types of flat CFRP panels (a reference one and two modified ones) for the LS test, commercial carbon–epoxy prepreg was used in the form of fabrics containing 36 wt.% of epoxy resin. Each panel was made from 16 prepreg layers using the out-of-autoclave method described above and the lay-up was as follows: [45/0/–45/90]_2S_. In the case of modified CFRP panels, expanded copper foil (ECF) and Tuball paper were laid on the outer surface of the prepregs stack. The curing process was then conducted according to the prepreg manufacturer’s recommendation. After the curing process, the flat panels were removed from the vacuum bag and cut into dimensions of 300 mm × 300 mm. Table 3 shows the designation and thickness of the manufactured CFRPs.

The expanded copper foil was supplied by Dexmet Corporation, Wallingford, CT, USA. The areal weight and the thickness of ECF were 141 g/m^2^ and 76 µm, respectively.

### 2.2. Measurement Methods

The surface electrical conductivity was measured using a Keithley 6517B Electrometer/High Resistance Meter (Tektronix, Beaverton, OR, USA) equipped with 8009 Test Fixture. The measuring cell was equipped with conductive electrodes made of rubber, which ensured perfect contact between the electrodes and the sample tested. Therefore, no additional conductive agent such as a silver paste or carbon tape was required. Before testing, five samples with dimensions of 75 mm × 75 mm were cleaned with acetone to remove dust and dirt. The test was carried out according to ASTM D-257, where the voltage applied was 1V, the time of the test was 15 s, and the number of readings to storage was adjusted to 5.

The volume electrical conductivity was tested through the laminate thickness in Z-direction. In this case, a Keithley 6221/2182A nanovoltmeter (Tektronix, Beaverton, OR, USA) and a direct current (DC) source were used. Unlike in the surface conductivity set, the device was equipped with copper electrodes. Therefore, it was necessary to use a conductive silver paste to ensure good contact between the sample and the electrodes. The thickness of the sample determined the distance between electrodes. The samples for these tests were 15 mm × 15 mm, and they were also cleaned with acetone and cut from different sections of the produced composite panels.

The quality of the manufactured laminates was controlled using a TM 3000 Hitachi, Tokyo, Japan scanning electron microscope (SEM). Before microstructure observations, the 15 mm × 15 mm samples were ground and polished, and then a thin layer of gold and palladium was sputtered on their surface. In order to observe the interface between the Tuball paper and CFRP, as well as the distribution of nanotubes in the Tuball paper, microstructure observations were carried out using a high-resolution SEM Hitachi SU70, Japan. First, the samples were embedded in conductive resin in order to secure edges of the samples composed of laminate and Tuball paper during grinding and polishing process, which used papers of four types of graininess: 240, 800, 1200 and 2400. The samples were then coated with a 3 nm Au–Pd electroconductive layer using 1.5 kV voltage, 10 mA current and 80 s time. SEM investigations were conducted using 5 kV accelerating voltage in secondary electron (SE) mode.

**Figure 1:**
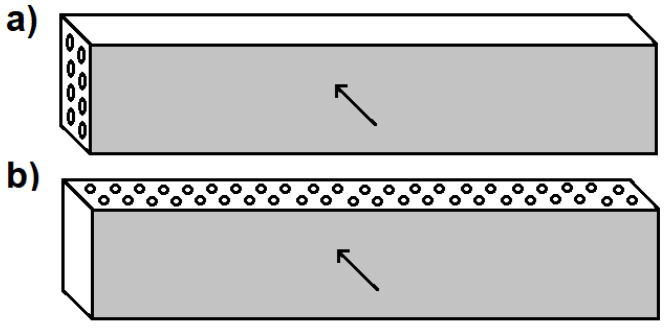
Method of performing impact resistance tests, striking edge (**a**) perpendicular and (**b**) parallel to carbon fibres.

To examine the effect of Tuball paper on impact resistance, the impact tests were applied according to PN-EN ISO 179-1 and carried out in two directions against the carbon fibres, as presented in Figure 1. Figure 1a features a Charpy hammer (Zwick Roell RKP 450, Zwick Roell Group, Ulm, Germany) with 300 J impact energy used in one direction while Figure 1b shows a CEAST Charpy Resil 5,5 with 4J impact energy applied in another direction. Six 75 mm × 15 mm samples without notch were prepared from each laminate according to PN-EN ISO 179-1. The surface of the laminates fracture was observed using a TM 3000 Hitachi, Japan scanning electron microscope.

The effect of Tuball paper on the stiffness of the manufactured CFRPs and the glass transition temperature were analyzed using dynamic mechanical analyser DMA Q800 (TA Instruments, New Castle, DE, USA). Samples of 60 mm × 10 mm were bent in a dual cantilever holder, from 0 °C to 250 °C with heating rate of 2 °C/min. All laminates were bent at a frequency of 1 Hz with an amplitude of 20 µm.

**Figure 2:**
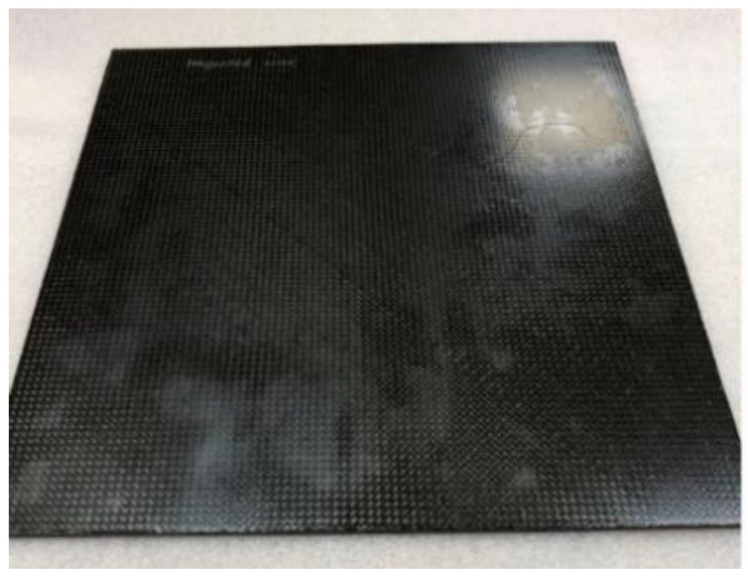
Reference test panel (L_ref). Dimensions: 300 mm × 300 mm.

**Figure 3:**
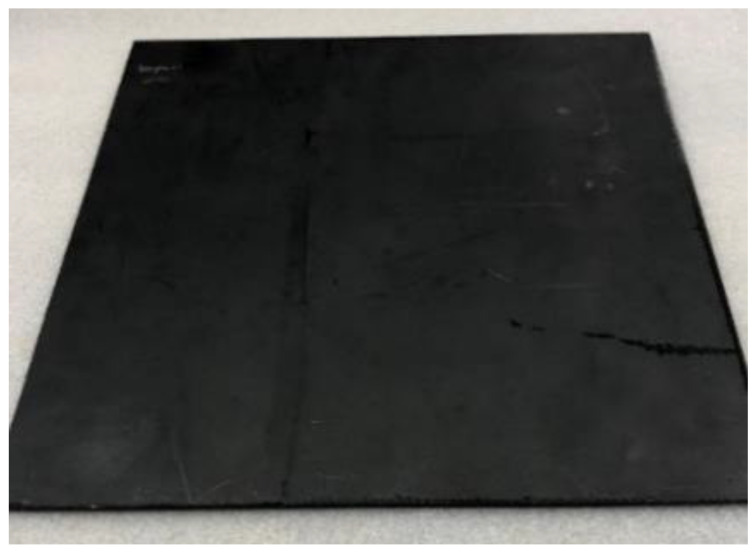
Test panel with Tuball paper (L_TP). Dimensions: 300 mm × 300 mm.

**Figure 4:**
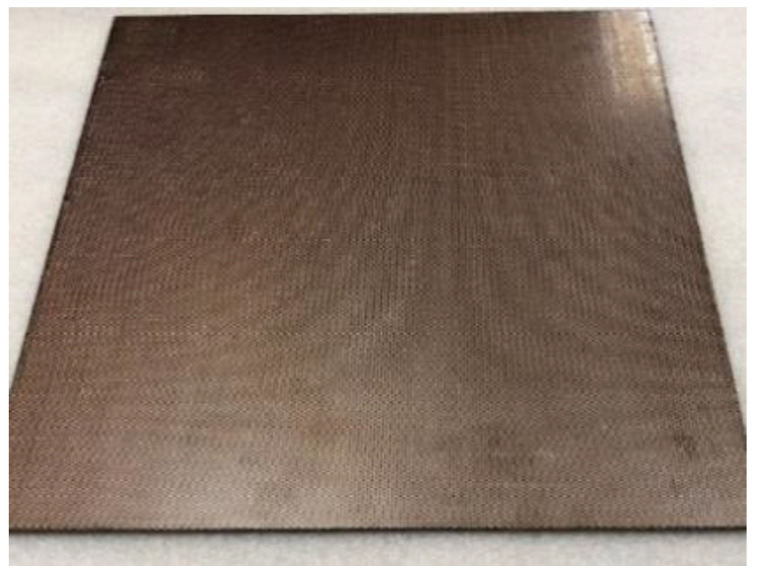
Test panel with ECF (L_ECF). Dimensions: 300 mm × 300 mm.

The LS test was performed by PolyTech Test and Validation in Denmark. The test was intended to demonstrate the performance of each of the test panels when subjected to the high current physical damage test, simulating a direct lightning strike to an aircraft structure located in zone 2A, according to ARP5414. The test was performed according to the method T02 Direct Effects—Structural method described in MIL-STD-1757A/Arc Entry Test method described in SAE ARP 5416. All test samples: the reference panel (No.1), the panel with Tuball paper (No.2) and the panel with ECF (No.3), have been exposed to energy-level defined values in MIL-STD-1757A/SAE (0.20–0.30 MJ/Ω) when being subjected to the D-component. Then, the test panel was subjected to charge simulating the B/C component. The test equipment simulating the D-component, is an impulse current generator producing an oscillating current waveform which represents the restrike current according to MIL-STD-1757A/subsequent stroke current according to SAE ARP 5412. The test equipment simulating the B/C components is a DC-charge storage bank providing a DC current, which represents the intimidate and continuing currents of the B/C components according to MIL-STD-1757A/SAE ARP 5412. The photos of panels before the test are presented in Figure 2, Figure 3 and Figure 4.

The test sequence (D component and B/C components) for the panels is described in Table 4 and Table 5, respectively.

**Figure 5:**
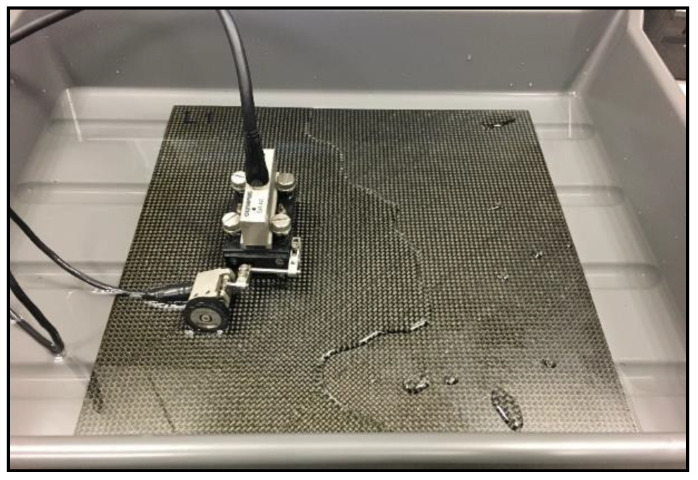
View of the immersion method.

Ultrasonic testing (UT) was performed using Olympus MX2 flaw detector and 5 MHz Phased Array probe with a delay line made of Aqualene elastomer. A contact pulse-echo technique was used and the tested parts were immersed in water, as shown in Figure 5.

A linear scan in a normal direction was performed with an 8-element active aperture. The results were obtained as B-and C-Scans. On C-Scan defects are visible as areas of warmer colors (yellow, orange, red), while areas without defects are visible as colder colors (blue, white).

## 3. Results and Discussion

### 3.1. Electrical Conductivity

**Figure 6:**
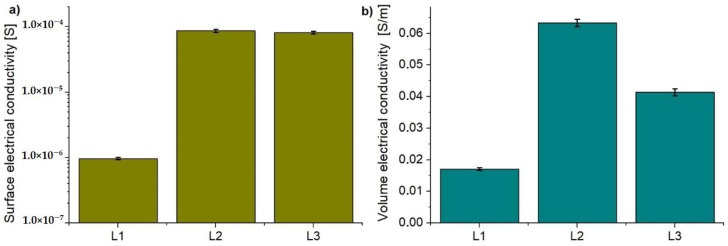
Electrical conductivity of CFRP panels: (**a**) surface, (**b**) Z-direction (through the thickness).

The results of surface electrical conductivity of the CFRPs modified with Tuball paper in comparison with unmodified CFRP are presented in Figure 6a. For the reference laminate, a value of 9.69 × 10^−7^ S was observed while for modified L2 and L3 laminates, surface electrical conductivity was 8.65 × 10^−5^ S and 8.15 × 10^−5^ S, respectively. The introduction of Tuball paper as the top layer of the laminate improved the surface electrical conductivity by two orders of magnitude. A slight difference in the improvement of electrical conductivity by application of TP1 (L2) in comparison with TP2 (L3) was observed due to the higher content of SWCNTs in TP1.

The modification of laminates by application of Tuball paper on their top also resulted in an increase in the volume conductivity in Z-direction, as shown in Figure 6b. A threefold and twofold increase in electrical conductivity was achieved for L2 and L3 laminates, respectively. As reported in the literature, introduction of BPs based on MWCNTs between each layer of the reinforcement allows for obtaining an increase in electrical conductivity by 5–6 times compared to the reference laminate [38,39]. This results from the formation of conductive paths between the layers of carbon fibres [40]. The introduction of conductive Tuball paper only on the top improved conductivity through the thickness of the laminate due to the elimination of an insulating top epoxy layer, which significantly increases the resistivity of CFRPs. Although the thickness of TP1 (L2) is almost two times lower and its areal weight is slightly lower, its conductivity is higher due to the higher content of SWCNTs. Consequently, higher volume electrical conductivity of L2 in comparison with L3 was achieved. The influence of Tubal paper’s thickness on electrical conductivity will also be discussed in Section 3.2, along with confirmed images of the microstructure.

### 3.2. Microstructure

**Figure 7:**
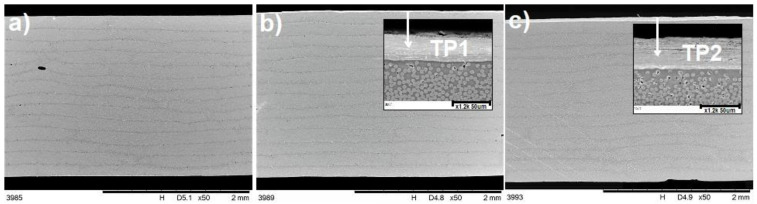
Microstructure of laminates cross-section: (**a**) L1, (**b**) L2, (**c**) L3. Arrows show the layer of Tuball paper.

The microstructure of the manufactured laminates is presented in Figure 7, where layers of carbon fibres are visible, as well as the top layer of Tuball papers (Figure 7b,c). It was observed that the application of Tuball paper on the surface of CFRPs did not affect the quality of the produced laminates. The difference in thickness of Tuball paper layers is also visible. SEM observations shown in Figure 7b,c, as well as 8 proved that TP1 is thinner, denser and therefore less amount of insulating epoxy resin has infiltrated its structure. That is why in the case of TP1 the increase in electrical conductivity is higher.

**Figure 8:**
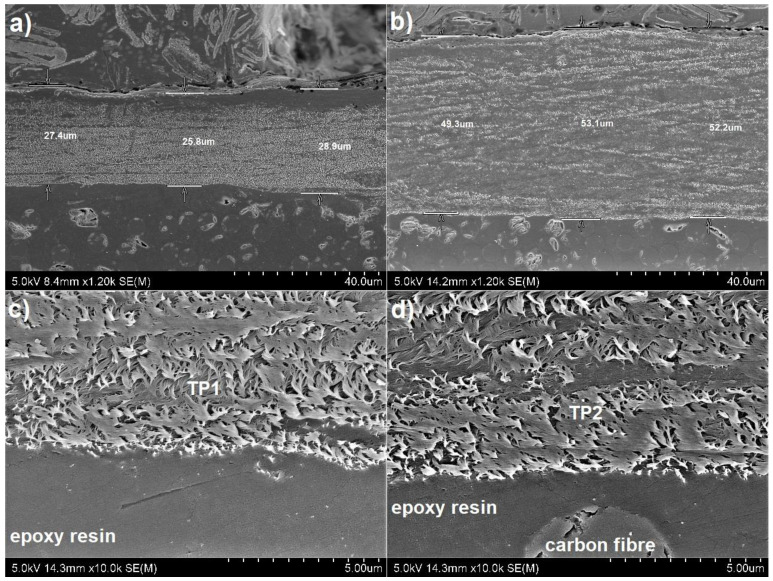
Microstructure of the cross-section of laminates with Tuball paper on the top: (**a**) and (**c**)—L2; (**b**) and (**d**)—L3. The arrow shows epoxy resin inside the layer.

Microstructure observations of the compatibility between Tuball paper and epoxy based laminates are shown in Figure 8. Figure 8c,d confirm very good infiltration of Tuball paper by epoxy resin and denser structure of TP1. No delamination between Tuball paper and CFRP has been observed. The lack of delamination at the Tuball paper/CFRP interface ensures good electrical conductivity. Improvement of electrical conductivity was also observed in the literature in the case of buckypaper based on MWCNTs [34].

### 3.3. Impact Resistance

**Figure 9:**
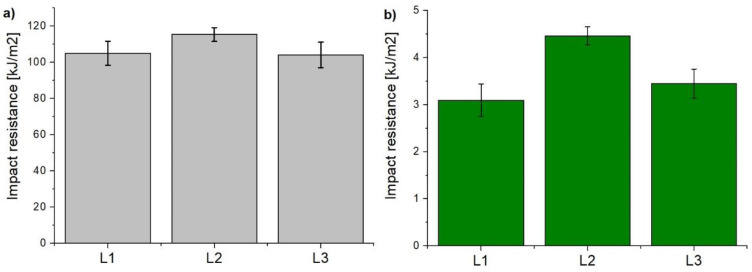
Impact resistance of analyzed laminates (**a**) perpendicular and (**b**) parallel to carbon fibres.

Charpy impact tests were carried out to compare the impact resistance of CFRPs modified with Tuball papers with that of the reference laminate. The results are presented in Figure 9. Considering the anisotropy of CFRPs, the tests were carried out in two directions—perpendicular (Figure 9a) and parallel (Figure 9b) to the carbon fibres. In the perpendicular direction these are mainly carbon fibres that carry the load, while in the parallel direction—practically only epoxy resin performs that function. Having analyzed the results, it was found that the introduction of Tuball paper on the top of the laminates improved the CFRPs impact resistance. This is due to the very good elastic properties of SWCNTs which are able to sustain much higher torsional, tensile or compressive stresses than other materials, especially epoxy resin [40]. Clearly higher impact resistance was observed in L2 laminate finished with the TP1 Tuball paper due to higher concentration of SWCNTs, although the paper thickness was lower. It is also reported in the literature that the presence of BP based on MWCNTs as the top layer of the CFRPs strengthened it, resulting in an increase in impact energy absorption and enhancement of matrix fracture toughness [41].

**Figure 10:**
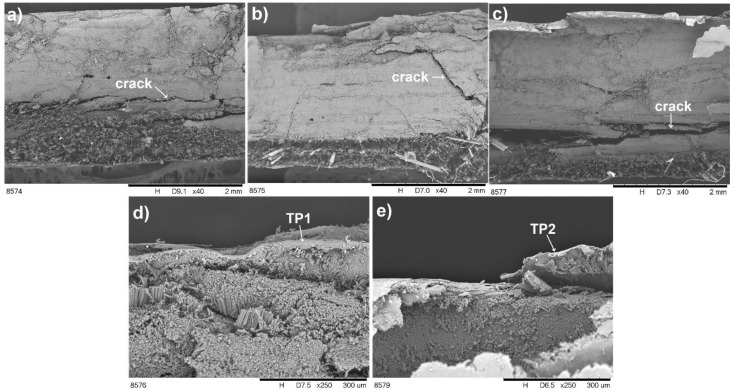
Microstructure of laminates after impact tests: (**a**) L1, (**b**,**d**) L2, (**c**,**e**) L3.

In order to explain the improvement of impact resistance, microstructure observations were carried out after impact tests and Figure 10 presents the results obtained in perpendicular direction. It can be seen in Figure 10a–c that the laminate L2 exhibits the lowest damage of its structure, which translates into the highest impact resistance. Additionally, for the L3 laminate, TP delamination was observed after the impact test (Figure 10e). In contrast, no delamination was recorded for the L2 laminate (Figure 10d), which explains the highest impact resistance obtained for this composite.

Taking into account the results of electrical conductivity and impact resistance, Tuball paper designed as TP1 was selected for lightning strike protection tests.

### 3.4. Dynamic Mechanical Analysis

**Figure 11:**
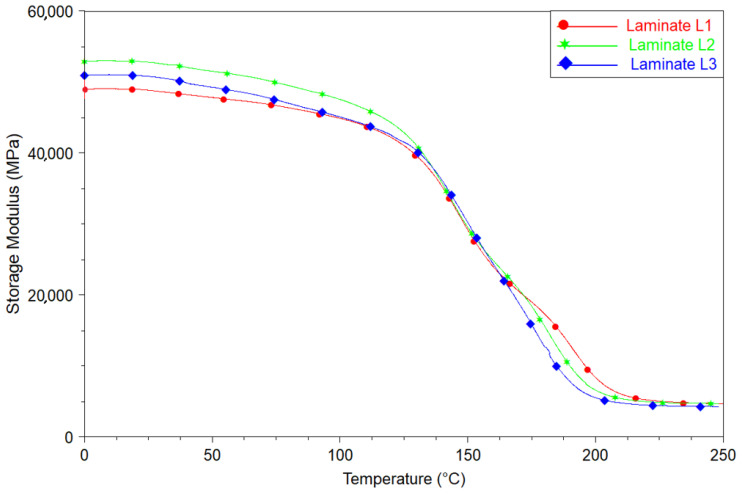
Storage modulus of reference (L1) and modified (L2, L3) laminates.

The introduction of a Tuball paper layer on the top of CFRPs leads to a slight change in the dynamic mechanical properties of CFRPs, as shown in Figure 11. It can be observed that the application of buckypaper led to an increase in the storage modulus in the glassy state [42]. In addition, the presence of SWCNTs on the top of the composite caused an increase in the glass transition temperature (Tg), starting from 124 °C for reference L1 up to 130 and 126 °C for L2 and L3, respectively, which were determined using TA Universal Analysis software. The increase in Tg is caused by the limitation of epoxy chains’ mobility in the presence of carbon nanotubes. The increase in the glassy storage module of epoxy resin and limitation in the mobility of epoxy chains as a result of the presence of MWCNTs, are behaviors referred in the literature [43,44]. Due to very good infiltration of Tuball paper through epoxy resins, it was possible to achieve this effect for L2 and L3 laminates. The results confirmed that SWCNTs act in the epoxy matrix in exactly the same way as MWCNTs. Moreover, differences observed in the dynamic properties of L2 and L3 laminates, are caused by different content of SWCNTs in Tuball papers. These may also be affected by SWCNTs dispersion in the Tuball paper, as it was found in the case of BP and MWCNTs [45].

### 3.5. Ultrasonic Tests after a Lightning Strike

**Figure 12:**
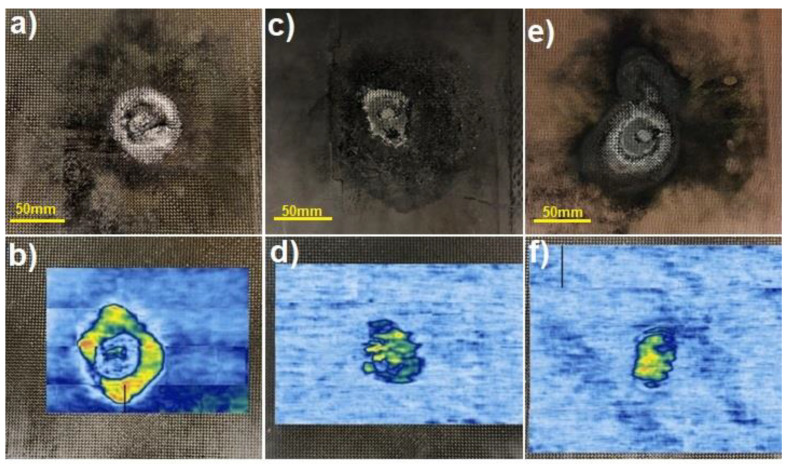
Images of composites flat panels after a lightning strike (upper row) and ultrasonic C-Scan visualization (lower row) for: (**a**,**b**) L_ref, (**c**,**d**) L_TP and (**e**,**f**) L_ECF.

Images of CFRP flat panels after lightning strike tests are shown in Figure 12a,c,e. The damaged area is visible in each case, even if ECF is applied on the top of CFRP. Evaluation of the damaged surface and the depth of the delamination ultrasonic testing was performed as an efficient method of detecting defects caused by a lightning strike. Application of Phased Array probes allows for immediate analysis of received signals in the form of B-Scan and C-Scan visualizations, making detection and evaluation of defects very effective. B-Scan represents the relationship between the ultrasonic signal amplitude and index axis (Y coordinate), while C-Scan determines the dependence of the ultrasonic signal amplitude (or sample thickness) on X and Y coordinates of the Phased Array probe location. Usually, defects are visible as areas of warmer colors (yellow, orange, red), while not defected area are visible as colder colors (blue, white). The methodology using Phased Array technique enabled fast volumetric testing of composite panels after a lightning strike, precise localization of defects and efficient handling of output data. After LSP tests, composite panels were scanned using the ultrasonic phased array method and Figure 12b,d,f present C-scans of the surface after the electric impact.

The introduction of both Tuball paper and ECF on the top of CFRP reduced the laminate surface damage, as evidenced by a much smaller area of warmer colors (yellow, orange, red). In both CFRPs with modified surface less damage of the structure was caused by faster electrical discharge. The electrical discharge is accompanied by Joule heat, and the temperature inside the composite exceeds the temperature of the thermal stability of the epoxy resin, which results in degradation of the polymer matrix [46]. For the reference laminate, the delamination dimensions were about 70 mm horizontally and 85 mm vertically. Modification of CFRP by Tuball paper reduced the dimensions of the surface damage to 45 mm horizontally and 55 mm vertically. The smallest damaged surface was observed in the laminate modified by ECF, in which the size of the delamination reached the values of 32 mm horizontally and 45 mm vertically. However, this destruction is more intense in green and yellow colors than in the case of laminate modified by Tuball paper.

**Figure 13:**
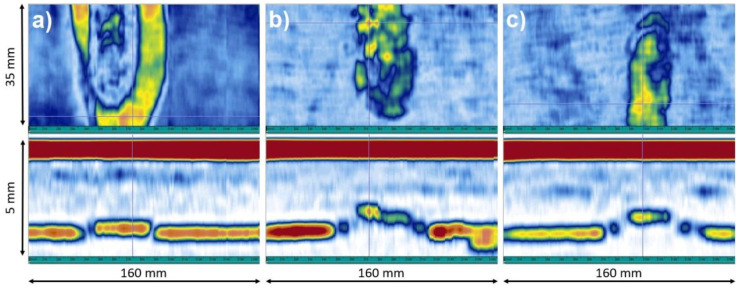
Images of samples after a lightning strike and ultrasonic tests for (**a**) L_ref, (**b**) L_TP and (**c**) L_ECF, where C-Scan (top) and B-Scan (bottom) visualization.

In Figure 13a–c, the results of C-Scan and B-Scan are collected for reference CFRP and CFRPs modified with ECF and Tuball paper, respectively.

Having analyzed the B-scans, it can be concluded that despite the most significant area of surface damage confirmed by the C-scan, which was obtained for the reference laminate, delamination in the reference composite material occurred at the smallest depth—0.2 mm, measured from the impacted surface. However, in modified laminates—L_TP and L_ECF, it was 0.85 mm and 0.6 mm respectively. The results confirmed that the mechanism of discharging of electrical charge is different when copper mesh or Tuball paper is applied on the top of CFRPs. The Tuball paper acts similarly to ECF as LSP in CFRPs and taking into account the mass saving it seems to be a promising alternative to the copper mesh.

## 4. Conclusions

The present work investigated the effect of Tuball papers of different SWCNTs content, thickness and areal weight on the electrical and mechanical properties of CFRPs. The studies aimed to select the best material for lightning strike protection of aircraft structures as a potential replacement of the commonly used but heavy copper mesh. For this purpose, flat CFRP panels were produced by the out-of-autoclave method and the surface of two of them was modified using Tuball papers. In the case of surface electrical conductivity, an increase of two orders of magnitude was achieved for both the L2 and L3 panels. In the case of volume conductivity, the L2 panel exhibits electrical conductivity three times higher than the reference one, while L3 shows only twice as high conductivity as a reference one, which is caused by higher concentration of SWCNTs in TP1 Tuball paper. It was observed that the application of Tuball paper during the CFRPs manufacturing did not affect the quality of the produced laminates, which are free of voids and air bubbles. Moreover, Tuball paper is well infiltrated by epoxy resin. Microscopic observations also confirmed good adhesion of Tuball paper to CFRP. Investigation showed that the glass transition temperature (Tg) increases by several degrees when Tuball paper is applied on the top of CFRPs. Higher Tg was observed for TP1 due to higher concentration of SWCNTs. The increase in Tg is caused by the limitation of epoxy chains’ mobility in the presence of carbon nanotubes. This phenomenon also leads to the strengthening of epoxy resin by the carbon nanotubes, which was confirmed by the results of impact and DMA tests. SWCNTs strengthened epoxy matrix, resulting in an increase in impact energy absorption and enhancement of fracture toughness, as well as an increase in storage modulus. The higher the concentration of SWCNTs in Tuball paper, the more significant the improvement of electrical and mechanical properties. The ultrasonic investigation of the CFRP flat panels after lightning strike tests proved that a thin layer of Tuball paper protects the composite as effectively as copper mesh. The area and the depth of the delamination were similar in both cases, and significantly different when compared to CFRP without any protection. Due to the significant mass saving, Tuball paper can be considered as an alternative to copper mesh in airplane applications.

## Figures and Tables

**Table 1:** Characteristics of Tuball papers used.

Tuball Paper Designation	Content of SWCNT (wt.%)	Thickness (µm)	Areal Weight (g/m^2^)
TP1	90	27.4 ± 3.11	41.5
TP2	75	51.5 ± 7.23	45.0

**Table 2:** Characteristics of the laminates manufactured for electrical and mechanical tests.

Materials	CFRP Designation	Thickness (mm)
CFRP reference	L1	2.29 ± 0.004
CFRP + TP1	L2	2.31 ± 0.007
CFRP + TP2	L3	2.33 ± 0.011

**Table 3:** Characteristics of composite panels manufactured for LS tests.

Material	CFRP Designation	Thickness (mm)
CFRP reference	L_ref	3.22
CFRP + Tuball paper	L_TP	3.28
CFRP + expanded copper foil	L_ECF	3.26

**Table 4:** T02, 2A–D Component test sequence.

Test Panel	Test ID	Peak Current (kA)	Specific Energy (MJ/Ω)	DC Charge (C)	Simulating
L_ref	1.1	100 ± 10%	0.25 ± 20%	-	D-component
L_TP	2.1	100 ± 10%	0.25 ± 20%	-	D-component
L_ECF	3.1	100 ± 10%	0.25 ± 20%	-	D-component

**Table 5:** T02, 2A–B/C Component.

Test Specimen	Test ID	Peak Current (kA)	Specific Energy (MJ/Ω)	DC Charge (C)	Simulating
L_ref	1.2	-	-	10 + 200 + 20%	B/C component
L_TP	2.2	-	-	10 + 200 + 20%	B/C component
L_ECF	3.2	-	-	10 + 200 + 20%	B/C component

## Data Availability

The data presented in this study are available on request from the corresponding author.
